# Physicochemical, Antioxidant, and Antimicrobial Properties of Three Medicinal Plants from the Western Part of the Rhodope Mountains, Bulgaria

**DOI:** 10.3390/life13122237

**Published:** 2023-11-21

**Authors:** Albena Parzhanova, Velichka Yanakieva, Ivelina Vasileva, Maria Momchilova, Dimitar Dimitrov, Petya Ivanova, Yulian Tumbarski

**Affiliations:** 1 Department of Food Technologies, Institute of Food Preservation and Quality, Agricultural Academy, 154 Vasil Aprilov Blvd., 4003 Plovdiv, Bulgaria; albenadsp@abv.bg (A.P.); masha821982@abv.bg (M.M.); 2 Department of Microbiology, University of Food Technologies, 26 Maritsa Blvd., 4002 Plovdiv, Bulgaria; yanakieva_vili@abv.bg; 3 Department of Organic Chemistry and Inorganic Chemistry, University of Food Technologies, 26 Maritsa Blvd., 4002 Plovdiv, Bulgaria; ivelinavas@abv.bg; 4 Department of Selection, Enology and Chemistry, Institute of Viticulture and Enology, Agricultural Academy, 1 Kala Tepe Str., 5800 Pleven, Bulgaria; dimitar_robertov@abv.bg; 5 Department of Biochemistry and Molecular Biology, University of Food Technologies, 26 Maritsa Blvd., 4002 Plovdiv, Bulgaria; petia_ivanova_georgieva@abv.bg

**Keywords:** antioxidants, herbs, phenolic compounds, extracts, antimicrobial activity

## Abstract

The present study examined the physicochemical, antioxidant, and antimicrobial properties of three medicinal plants: thyme (*Thymus callieri* Borbás ex Velen), cotton thistle (*Onopordum acanthium* L.), and hawthorn fruit (*Crataegus monogyna* Jacq.) from the Western Rhodope Mountains, Bulgaria. The first stage determined the physicochemical characteristics (moisture, ash, carbohydrates, proteins, and vitamin C) of the three herbs. The second stage investigated four types of extracts (aqueous, oil, methanolic, and ethanolic) of each herb and evaluated their total phenolic content, the presence of phenolic compounds (flavonoids and phenolic acids), their antioxidant activity, and antimicrobial properties. Thyme was characterised by the highest ash, protein, and vitamin C content (6.62%, 11.30%, and 571 mg/100 g, respectively). Hawthorn fruit showed the highest moisture and carbohydrate content (8.50% and 4.20%, respectively). The 70% ethanolic extracts of the three herbs exhibited the highest levels of phenolic compounds and, consequently, pronounced antioxidant activity, compared to the other three types of extracts. The aqueous, oil, methanolic, and ethanolic thyme extracts demonstrated the highest total phenolic content—TPC (27.20 mg GAE/g, 8.20 mg GAE/g, 31.70 mg GAE/g, and 310.00 mg GAE/g, respectively), compared to the extracts of the other two plants. These results were consistent with the highest antioxidant activity of the thyme extracts determined using the 2,2-diphenyl-1-picrylhydrazyl (DPPH) radical scavenging assay, the oxygen radical absorbance capacity (ORAC) assay, and the hydroxyl radical averting capacity (HORAC) assay (except for the oil extract examined using the DPPH method). The results from the high-performance liquid chromatography (HPLC) analysis revealed that the flavonoid quercetin-3-ß-glucoside had the highest concentration in thyme (374.5 mg/100 g), while myricetin dominated in the cotton thistle (152.3 mg/100 g). The phenolic acid content analysis showed prevalence of rosmaric acid in the thyme (995 mg/100 g), whereas chlorogenic acid was detected in the highest concentration in the cotton thistle and hawthorn fruit (324 mg/100 g and 27.7 mg/100 g, respectively). The aqueous, methanolic, and ethanolic extracts showed moderate to high antibacterial potential but limited antifungal activity. None of the oil extracts inhibited the test microorganisms used in the study.

## 1. Introduction

In recent years, numerous scientific studies have revealed that the consumption of plant foods, including wild herbs and fruits, is of great importance for the prevention and treatment of various diseases, as well as for enhancing vitality and restoring good health due to their high nutritional value and healing properties [1,2,3,4]. This fact has been confirmed scientifically, showing that medicinal plants are valuable sources of vitamins, fibres, and polyphenolic compounds present in their tissues. Polyphenols are regarded as compounds with significant antioxidant potential, but they also determine the biological activities and health benefits of medicinal plants by reducing the risk of developing cardiovascular diseases, diabetes, osteoporosis, neurodegenerative disorders, and cancer [5,6,7,8,9,10,11,12].

Bulgaria is located in southeastern Europe, which has been divided into five climatic zones (temperate continental, transitional continental, continental Mediterranean, Black Sea, and mountainous) depending on the influence of certain climatic factors. Each of these zones is characterised by great soil and botanical diversity. According to some recent reports, more than 700 species (approximately 20% of the Bulgarian flora) are recognised as herbs, most of them being wild plants growing in the mountainous regions of the country [13].

**Figure 1:**
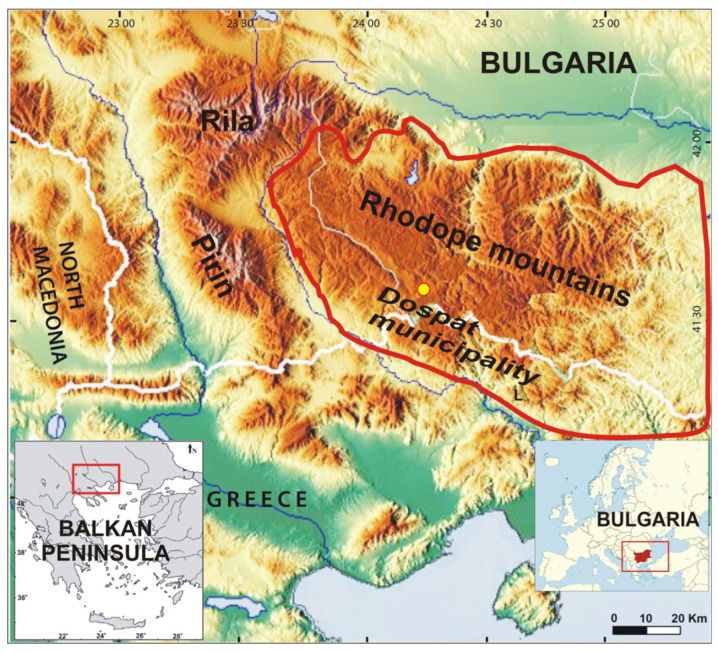
The Rhodope Mountains.

The Rhodope Mountains, or the Rhodopes, are the largest mountain range in Bulgaria, located in the southern part of the country (Figure 1). The Rhodopes are divided into two parts: western (average altitude of 1098 m) and eastern (average altitude of 329 m). Mountainous climate prevails in the higher altitude regions of the western Rhodopes, transitional continental climate dominates the lower altitude regions, while the southern parts are characterised by Mediterranean climate. A study by Zahariev et al. [14] revealed that the medicinal plants in the Rhodopes included 714 species belonging to 393 genera and 101 families. The largest part of this area has been designated as a protected area within the NATURA 2000 European ecological network aimed at the protection of rare and threatened habitats, plants, and animals. The medicinal plants are found mainly in grasslands and meadows, but they face the risk of extinction due to negative environmental changes. The most common medicinal plants in the Rhodope Mountains belong to the *Asteraceae*, *Lamiaceae*, *Rosaceae*, *Amrillydaceae*, *Crassulaceae*, *Plantaginaceae*, *Oleaceae,* and *Solanaceae* families [15]. Although Bulgaria is the second largest European exporter and one of the world’s leading exporters of herbs, the information about the botanical diversity, the phytochemical composition, and the biological properties of the wild plants growing in mountainous regions, the Rhodope Mountains in particular, is still very limited [13].

Hawthorn (*Crataegus monogyna*) is a plant of the *Rosaceae* family widely used in medicine and in culinary practice. Hawthorn fruits have been found to contain vitamin C, sugars, organic acids, phenols, flavonoids, and anthocyanins that contribute to their red, purple, or blue colour [16,17,18]. They are rich in minerals, i.e., Na, K, Ca, P, Al [19], Mg, Cu, Zn, and Fe [20]. Therefore, they are used for the prevention and treatment of cardiovascular diseases, cancer, diabetes, asthma, and nephritis, as well as for improving memory [21]. According to Tadić et al. [22], extracts of dried hawthorn (*Crataegus monogyna* Jacq., *C. oxyacantha* L. and *C. laevigata*) can be used as an anti-inflammatory, gastroprotective and, antimicrobial agent. Kostić et al. [23] found that acetone extracts of dried hawthorn (*C. oxyacantha* L.) demonstrated antioxidant activity. The same finding was confirmed by Ziouche et al. [24] who stated that hawthorn leaves and fruits were rich in polyphenolic compounds (rutin, quercetin, and isoquercetin) and exhibited significant antioxidant activity.

The *Thymus* genus belongs to the *Lamiaceae* family, which includes about 350 aromatic perennial species, indigenous to the Mediterranean region. The most popular species cultivated for culinary, medicinal, and ornamental purposes is *Thymus vulgaris*, or common thyme. The 21 *Thymus* species spread across Bulgaria include *Thymus serpyllum* (wild thyme) and *Thymus callieri* Borbás ex Velen, which have been found in different natural localities in Bulgaria. In the Rhodope Mountains, *T. callieri* Borbás ex Velen has been discovered in two locations near the town of Dospat, at an altitude of about 1200 m [25,26]. Studies by Djenane et al. [27] and Hyldgaard et al. [28] found that thyme contained polyphenols (flavonoids), organic acids, vitamin C, pigments, minerals (Ca, Fe, and Mn), and dietary fibres [26]. Thyme includes about 0.15–1.5% essential oil, the amount of which varies depending on the species, and up to 5% tannins and resinoid substances. Thyme essential oil extracted from the whole plant contains aromatic and volatile compounds (thymol, carvacrol, quercol, α-terpineol, L-borneol, L-cymol, L- and D-pinene, γ-terpene, caryophyllene, and linalool) and is known to possess antimicrobial and antioxidant properties [29,30].

Cotton thistle (*Onopordum acanthium* L.) is a biennial spiny herb of the *Asteraceae* family, widely spread worldwide (Europe, Asia, America, and Australia), including all over Bulgaria and growing mainly in dry and rocky areas [31]. In the cotton thistle leaves there are tannins, the sesquiterpene lactone arctiopicrin, saponins, and alkaloids. The flower baskets contain the polysaccharide inulin and an array of phytochemical compounds, including polyphenols, phenolic acids, flavonoids, acetylene, triterpenes [32], fatty acids (oleic, linoleic, linolenic, stearic, palmitic, pentadecanoic, and erucic), and phytosterols [33,34], which contribute to their antioxidant, anti-inflammatory, antimicrobial, diuretic, cardioprotective, and wound-healing properties.

Despite the great number of scientific publications on thyme, cotton thistle, and hawthorn, the information regarding the physicochemical characteristics and biological properties of some of these plants (for example, *Thymus callieri* Borbás ex Velen, which is among the rare species) is still very limited. Therefore, the aim of this research was to investigate the physicochemical, antioxidant, and antimicrobial properties of thyme, cotton thistle, and hawthorn fruit from the western part of the Rhodope Mountains in Bulgaria, which would contribute to the knowledge of the herbs in this region and their potential applications.

## 2. Materials and Methods

### 2.1. Materials

#### 2.1.1. Plant Material

In the current study, three medicinal plants, thyme, cotton thistle, and hawthorn fruit, were used. The herbs were harvested in the western part of the Rhodope Mountains, Bulgaria, in the June–August 2022 period, and identified according to the Herbarium Academiae Scientiarum Bulgariae (Table 1).

#### 2.1.2. Plant Extracts

The dried herbs were finely ground using a blender. Aqueous (infusion-type) extracts were obtained by pouring boiling distilled water over 10 g of the ground dry material at a hydromodulus of 1:20. The extraction was carried out for 30 min. After filtration, the extracts were stored at 4 °C for no more than 24 h until analysis. Oil extracts were obtained by pouring refined sunflower oil (Biser, Biser Oliva AD, Stara Zagora, Bulgaria) over 10 g of the ground dry material and then heating at 80 °C for 24–48 h with constant stirring. Methanolic and ethanolic extracts were obtained through maceration of 1 g of the ground dry material with 15 mL of methanol/70% ethanol (Sigma-Aldrich, Merck, Darmstadt, Germany); after that, the samples were stirred using a vortex shaker (V-1, Biosan, Riga, Latvia) for 10–15 s and left at room temperature for 48 h in darkness. The extracts obtained were filtered through filter paper and then stored at 4 °C for further analyses. Before use, the ethanol was vacuum-evaporated and the ethanolic extracts were diluted in methanol (which is not known to possess antimicrobial activity against the test microorganisms used).

#### 2.1.3. Test Microorganisms

Twenty microorganisms from the collection of the Department of Microbiology at the University of Food Technologies, Plovdiv, Bulgaria were selected for the antimicrobial activity screening. They included six gram-positive bacteria (*Bacillus subtilis* ATCC 6633, *Bacillus amyloliquefaciens* 4BCL-YT, *Staphylococcus aureus* ATCC 25923, *Listeria monocytogenes* NBIMCC 8632, *Enterococcus faecalis* ATCC 19433, and *Micrococcus luteus* 2YC-YT), six gram-negative bacteria (*Salmonella enteritidis* ATCC 13076, *Salmonella typhimurium* NBIMCC 1672, *Klebsiella pneumonia* ATCC 13883, *Escherichia coli* ATCC 25922, *Proteus vulgaris* ATCC 6380, and *Pseudomonas aeruginosa* ATCC 9027), two yeasts (*Candida albicans* NBIMCC 74 and *Saccharomyces cerevisiae* ATCC 9763), and six fungi (*Aspergillus niger* ATCC 1015, *Aspergillus flavus*, *Penicillium chrysogenum*, *Rhizopus* sp., *Mucor* sp.—plant isolates, and *Fusarium moniliforme* ATCC 38932).

*B. subtilis*, *B. amyloliquefaciens*, and *M. luteus* were cultured on LBG agar at 30 °C for 24 h, while *S. aureus, L. monocytogenes, L. innocua, E. faecalis, E. faecium, S. enteritidis, Klebsiella* sp., *E. coli, P. vulgaris,* and *P. aeruginosa* were cultured on LBG agar at 37 °C for 24 h. The *C. albicans* yeast was cultured on MEA at 37 °C, whereas *S. cerevisiae* was cultured on MEA at 30 °C for 24 h. The A. niger, *A. flavus, Penicillium* sp., *Rhizopus* sp., and *F. moniliforme* fungi were grown on MEA at 30 °C for 7 days or until sporulation.

#### 2.1.4. Culture Media

Luria-Bertani agar medium with glucose (LBG agar). LBG agar was used for the cultivation of the test bacteria. A quantity of 50 g of LBG-solid substance mixture was dissolved in 1 L of deionised water, pH 7.5 ± 0.2.

Malt extract agar (MEA). MEA was used for the cultivation of the test yeasts and fungi. A quantity of 50 g of the MEA-solid substance mixture was dissolved in 1 L of deionised water, pH 5.4 ± 0.2.

Both culture media were prepared in accordance with the manufacturer’s (Scharlab SL, Barcelona, Spain) instructions and autoclaved at 121 °C for 20 min before use.

### 2.2. Methods

#### 2.2.1. Physicochemical Analyses

The physicochemical characteristics of the herbs investigated in the study, i.e., moisture content, ash content, carbohydrate, protein, and vitamin C content, were determined according to the following Bulgarian State Standards (Table 2).

#### 2.2.2. Total Phenolic Content

The total phenolic content (TPC) was assessed using the method of Ivanov et al. [40]. The reaction mixture was prepared using 1 mL of Folin–Ciocalteu reagent (Sigma-Aldrich, Merck, Darmstadt, Germany), 0.8 mL of 7.5% sodium carbonate (Sigma-Aldrich, Merck), and 0.2 mL of the tested plant extract. The samples were left at room temperature for 20 min (in darkness). The absorbance was measured spectrophotometrically (Camspec M107, Spectronic-Camspec Ltd., Leeds, UK) at 765 nm against a blank (distilled water). The results were presented as mg gallic acid equivalent (mg GAE)/g.

#### 2.2.3. Antioxidant Activity

DPPH radical scavenging assay. The reaction mixture was prepared using 2.85 mL of DPPH reagent (2,2-diphenyl-1-picrylhydrazyl) and 0.15 mL of the tested plant extract. The samples were incubated at 37 °C for 15 min; then, the absorbance was measured at 517 nm against a blank (methanol). The antioxidant activity was expressed as µM Trolox equivalents (TE)/g of dry weight (dw) [40].

ORAC (Oxygen radical absorbance capacity) assay. ORAC was determined using the method described by Teneva et al. [41]. The reaction mixture was prepared using 170 µL of fluorescein disodium salt (FL), 20 µL of 2,2′-Azobis (2-amidinopropane) dihydrochloride, and 10 µL of the tested plant extract. The FL solution and the tested extract were incubated at 37 °C for 20 min in a microplate reader; then, the AAPH (dissolved in a phosphate buffer with pH 7.4 at 37 °C) was added and additionally incubated for 30 s. The fluorescence readings were taken at the end of each cycle after shaking. As a blank, 10 µL of a phosphate buffer was used. The standard curve was built using Trolox solutions. ORAC was measured using a plate reader FLUOstar OPTIMA (BMG Labtech, Ortenberg, Germany) at 485/520 nm. The results were expressed as µmol Trolox equivalents (µmol TE)/g of dw.

HORAC (Hydroxyl radical averting capacity) assay. HORAC was assessed using the method of Teneva et al. [41]. The FL solution (170 µL) and the tested extract (10 µL) were incubated at 37 °C for 10 min in the microplate reader. Next, 10 µL of hydrogen peroxide and 10 µL of Co (II) solution (15.7 mg of CoF_2_•4H_2_O and 20 mg of picolinic acid dissolved in 20 mL of distilled water) were added. The initial fluorescence was measured, after which the readings were taken every minute after shaking. As a blank, a phosphate buffer (pH = 7.4) was used. The standard curve was built using solutions of 100, 200, 600, 800, and 1000 µM gallic acid in a phosphate buffer. HORAC values were measured using a FLUOstar OPTIMA plate reader (BMG Labtech) at 485/520 nm. The results were expressed as µmol gallic acid equivalents (µmol GAE)/g of dw.

#### 2.2.4. High-Performance Liquid Chromatography (HPLC) Analysis of Phenolic Compounds

The phenolic compounds of the three investigated herbs were determined according to the method previously described by Teneva et al. [41] using a Nexera-i LC-2040C Plus UHPLC system (Shimadzu, Kyoto, Japan), equipped with a UV detector and a binary pump at 280 nm with sample injection volume of 20 µL. The phenolic compound separation was performed on an Agilent TC-C18 column (5 µm, 4.6 mm × 250 mm) at 25 °C, and the mobile phase included 0.5% acetic acid (A) and 100% acetonitrile (B) at a flow rate of 0.8 mL/min. The phenolic compounds were identified by comparing the retention times of unknown analytes with analytical grade standards. The results were expressed as mg/100 g of dw.

#### 2.2.5. Antimicrobial Activity Assay

The antimicrobial activity of the plant extracts was determined using the agar well diffusion method [42]. In the first stage of the experiment, bacterial, yeast, and fungal inocula were prepared. A Thoma bacterial counting chamber (Poly-Optik GmbH, Bad Blankenburg, Germany) was used for determination of the viable cells and fungal spore counts; after that, their final concentrations for inoculation were adjusted to 10^8^ cfu/mL (for bacterial/yeast cells) and to 10^5^ cfu/mL (for fungal spores). In the second stage, LBG/MEA, preliminarily melted and tempered at 45–48 °C, were inoculated with the bacterial/yeast/fungal inocula, the inoculated media were transferred into sterile Petri dishes (d = 90 mm) (Gosselin™, Hazebrouck, France) in 18 mL quantities, and they were allowed to harden for 1–2 h. The extracts were pipetted in 60 μL duplicates into pre-prepared wells (d = 6 mm) in the agar media. The Petri dishes were incubated under identical conditions according to the test microorganism type.

After incubation for 24/48 h, the antimicrobial activity of the extracts was determined by measuring the diameter of the inhibition zones (IZ) around the agar wells. The microorganisms with an IZ diameter of 18 mm or more were considered sensitive; those with an IZ diameter between 12 and 18 mm were moderately sensitive; and those where the IZ diameter was up to 12 mm or completely missing were resistant.

The antibiotics Ampicillin, Penicillin and Cyprofloxacin (against bacteria), and Fluconazole and Nystatin (against yeast and fungi) in 10 mg/mL concentrations were used as controls.

#### 2.2.6. Statistical Analysis

The results of the experiments run in triplicate were presented as mean values ± standard deviation (±SD). One-way analysis of variance (ANOVA) was performed using the Statgraphics Centurion statistical program, version XVI, 2009 (Stat Point Technologies, Inc., Warrenton, VA, USA). The mean differences were established using Fisher’s least-significant difference test for paired comparison at a significance level of *p* ≤ 0.05.

## 3. Results

The results of the physicochemical analyses of the three investigated herbs have been presented in Table 3.

The results show that thyme, cotton thistle, and hawthorn fruit had similar moisture content values. Regarding the ash content, thyme showed the highest value (6.62%), followed by cotton thistle and hawthorn fruit. Hawthorn fruit exhibited the highest carbohydrate content (4.2%), followed by thyme and cotton thistle. Out of the three herbs studied, thyme demonstrated the highest protein content (11.3%), while the lowest value was determined for hawthorn fruit. The highest vitamin C content was found in thyme (571 mg/100 g dw), followed by hawthorn fruit and cotton thistle.

### 3.1. Polyphenolic Content and Antioxidant Activity

As seen from the results presented in Table 4 and Table 5, the 70% ethanolic extracts of the three herbs demonstrated the highest total polyphenolic content (TPC) and antioxidant activity (determined using the DPPH, ORAC and HORAC methods) in comparison with the aqueous, oil, and methanolic extracts, which exhibited significantly lower values. Out of the three investigated herbs, the thyme extracts (aqueous, oil, methanolic, and ethanolic) were characterised by the highest total phenolic content and antioxidant activity, except for the oil extract examined through the DPPH assay. Consequently, the solvent type had a significant effect on the extract activity as organic solvents achieve better extraction of biologically active substances.

### 3.2. Flavonoids and Phenolic Acid Contents

Flavonoids are the polyphenolic compounds most abundant and widely distributed in plants. The results of the high-performance liquid chromatography analysis (HPLC) of the flavonoid content of the three investigated herbs have been presented in Table 6.

The flavonoid content of thyme was characterised by the highest amount of quercetin-3-β-glucoside (374.5 mg/100 g), followed by luteolin (73.8 mg/100 g), while apigenin and kaempferol were detected in significantly lower concentrations. The flavonoid content of cotton thistle was characterised by the highest amount of myricetin, followed by apigenin and kaempferol. In contrast, only quercetin-3-β-glucoside and a low amount of myricetin were presented in the flavonoid content of hawthorn fruit.

Regarding the phenolic acid content of the three plants determined using the HPLC method (Table 7), thyme demonstrated the highest presence of rosmaric acid (995 mg/100 g) and a significantly lower concentration of caffeic acid. The phenolic acid content of cotton thistle was represented by chlorogenic acid (324 mg/100 g) and lower amounts of neochlorogenic acid and gallic acid. The phenolic acid content of hawthorn fruit included chlorogenic acid, neochlorogenic acid, and caffeic acid.

### 3.3. Antimicrobial Activity

As seen from the results in Table 8 and Table 9, the aqueous, methanolic, and ethanolic extracts of the three herbs studied showed moderate to high inhibitory activity against gram-positive and gram-negative bacteria but limited antifungal potential. The 70% ethanolic extracts of the three herbs exhibited the highest antibacterial activity in comparison with the methanolic and aqueous ones. None of the oil extracts inhibited the test microorganisms used in the study (data not presented). The antimicrobial activity of all plant extracts was lower compared to the conventional antibacterial and antifungal antibiotics used as positive controls.

The aqueous extract of thyme demonstrated the highest inhibitory activity against *M. luteus* 2YC-YT, moderate activity against *L. monocytogenes* NBIMCC 8632, *S. enteritidis* ATCC 13076, and low inhibitory activity against *S. aureus* ATCC 25923 and fungi *P. chrysogenum* and *Rhizopus* sp. The aqueous extract of hawthorn fruit showed a moderate inhibitory effect on *L. monocytogenes* NBIMCC 8632, *E. faecalis* ATCC 29212, *M. luteus* 2YC-YT, and fungi *Rhizopus* sp. and a weak inhibitory effect on *B. subtilis* ATCC 6633, *P. aeruginosa* ATCC 9027, *E. coli* ATCC 25922, and fungus *P. chrysogenum*. The aqueous extract of cotton thistle demonstrated the highest inhibitory activity against *M. luteus* 2YC-YT, moderate activity against *L. monocytogenes* NBIMCC 8632, *E. faecalis* ATCC 29212, *S. enteritidis* ATCC 13076, and fungus *P. chrysogenum*, while its inhibitory effect on the *Rhizopus* sp. fungus was weak.

The methanolic extract of thyme had a moderate inhibitory effect on *B. subtilis* ATCC 6633, *S. enteritidis* ATCC 13076, *S. typhimurium* NBIMCC 1672, *K. pneumoniae* ATCC 13883, *E. coli* ATCC 25922, *P. aeruginosa* ATCC 9027, and *P. vulgaris* ATCC 6380. Its antimicrobial activity against *B. amyloliquefaciens* 4BCL-YT, *S. aureus* ATCC 25923, *L. monocytogenes* NBIMCC 8632, *E. faecalis* ATCC 29212, *M. luteus* 2YC-YT, yeasts *C. albicans* NBIMCC 74, *S. cerevisiae* ATCC 9763, fungi *A. niger* ATCC 1015, *P.chrysogenum*, *Rhizopus* sp., and *F. moniliforme* ATCC 38932 was low.

The methanolic extract of hawthorn fruit exhibited a moderate inhibitory effect only on *K. pneumoniae* ATCC 13883, while its inhibitory effect on *B. subtilis* ATCC 6633, *B. amyloliquefaciens* 4BCL-YT, *M. luteus* 2YC-YT, *S. typhimurium* NBIMCC 1672, *E. coli* ATCC 25922, *P. aeruginosa* ATCC 9027, *P. vulgaris* ATCC 6380, yeast *C. albicans* NBIMCC 74, and fungus *P. chrysogenum* was weak.

The methanolic extract of cotton thistle showed a moderate inhibitory effect on *P. aeruginosa* ATCC 9027 and *P. vulgaris* ATCC 6380. Its antimicrobial activity against *B. amyloliquefaciens* 4BCL-YT, *B. subtilis* ATCC 6633, *M. luteus* 2YC-YT, *S. typhimurium* NBIMCC 1672, *K. pneumoniae* ATCC 13883, *E. coli* ATCC 25922, yeast *C. albicans* NBIMCC 74, and fungus *P. chrysogenum* was low.

The ethanolic extract of thyme had high antimicrobial activity against *S. aureus* ATCC 25923 and *E. coli* ATCC 25922, moderate activity against *B. subtilis* ATCC 6633, *B. amyloliquefaciens* 4BCL-YT, *L. monocytogenes* NBIMCC 8632, *E. faecalis* ATCC 29212, *M. luteus* 2YC-YT, *S. enteritidis* ATCC 13076, *K. pneumoniae* ATCC 13883, *P. vulgaris* ATCC 6380, and *P. aeruginosa* ATCC 9027, and weak antimicrobial activity against *S. typhimurium* NBIMCC 1672, yeast *S. cerevisiae* ATCC 9763, and fungi *P. chrysogenum*, *Rhizopus* sp., and *F. moniliforme* ATCC 38932.

The ethanolic extract of hawthorn fruit showed a moderate inhibitory effect on *B. subtilis* ATCC 6633, *L. monocytogenes* NBIMCC 8632, *M. luteus* 2YC-YT, *P. aeruginosa* ATCC 9027, and *E. coli* ATCC 25922 and a weak inhibitory effect on *B. amyloliquefaciens* 4BCL-YT, *E. faecalis* ATCC 29212, *S. enteritidis* ATCC 13076, *S. typhimurium* NBIMCC 1672, *K. pneumoniae* ATCC 13883, and *P. vulgaris* ATCC 6380.

The ethanolic extract of cotton thistle demonstrated a strong inhibitory effect on *B. subtilis* ATCC 6633, *M. luteus* 2YC-YT, and *E. coli* ATCC 25922 and a moderate inhibitory effect on *S. aureus* ATCC 25923, *L. monocytogenes* NBIMCC 8632, *E. faecalis* ATCC 29212, *S. enteritidis* ATCC 13076, *S. typhimurium* NBIMCC 1672, *P. aeruginosa* ATCC 9027, and *K. pneumoniae* ATCC 13883. Its antimicrobial activity against *P. vulgaris* ATCC 6380, yeasts *C. albicans* NBIMCC 74, *S. cerevisiae* ATCC 9763, and fungi *A. niger* ATCC 1015, *P. chrysogenum*, *Rhizopus* sp., and *F. moniliforme* ATCC 38932 was low.

## 4. Discussion

### 4.1. Physicochemical Characteristics

The physicochemical characteristics of medicinal plants are important indicators of their quality. Different environmental conditions may influence their chemical composition [43]. For example, the protein content (14.9%) of *O. acanthium* L. originating from Turkey was higher in comparison with our results (8.33%) [44]. Results similar to ours were previously reported by Petkova et al. [45], who investigated *O. acanthium* L. from Bulgaria and determined moisture content of 7.9% and ash content of 5.8%. Similar physicochemical characteristics of thyme (*Thymus vulgaris* Linne) were reported by Balladin and Headley [46], who stated that the moisture content of thyme dried at different temperature modes varied between 10% and 12.5%, while the ash content ranged between 1.5% and 2.26%—values lower than our results. In contrast to our data, Shahar et al. [47] determined higher protein (21.39%) and carbohydrate (11.85%) amounts but lower ash content (2.73%) and vitamin C concentration (43.8 mg/100 g dw) in thyme (*Thymus serpyllum* L.) from the Kargil Ladakh district, India. According to Li et al. [48], the physicochemical characteristics of hawthorn fruit are usually influenced by the species and collection location (origin). Özcan et al. [49] established that postharvest handling and processing influenced the chemical composition of hawthorn (*Crataegus* spp.) fruit in Turkey. The authors obtained similar results to ours for moisture content (64.26%), ash content (2.28%) and crude protein content (2.48%). Mironeasa et al. [50] investigated the physicochemical characteristics of fresh wild hawthorn fruit and determined that the ash and protein contents were in agreement with our results (1.75% and 3.5%, respectively), but the fresh fruit showed significantly higher moisture and carbohydrate contents (69.14% and 24.81%, respectively). Yanar et al. [51] reported that vitamin C values in three Turkish wild hawthorn genotypes varied between 1.3 and 6 mg/100 g fw, which was significantly lower compared to our results for dried fruit (430 mg/100 g dw). Probably, the genetic variation among species also affects the content of these compounds.

### 4.2. Polyphenolic Content and Antioxidant Activity

In general, the yield of phenolic compounds from plants depends on certain extraction parameters like solvent concentration, extraction time, and temperature, which is why the described differences in the results of the different author collectives are observed. The extraction of polyphenols from plant material can be achieved using different solvent system [52]. According to Goli et al. [53], the extraction yield is dependent on the solvent and the method of extraction. Sun and Ho [54] reported that water and aqueous mixtures of ethanol or methanol are commonly used in plant extraction.

The differences in growth conditions in different parts of the world affect the content of polyphenolic compounds of the plants. Roby et al. [55] obtained results lower than ours on the total phenolic content of thyme (*T. vulgaris*) from Egypt. They measured values of 8.1, 7.3, 6.15, and 4.75 mg GAE/g dw for the methanolic, ethanolic, diethyl ether, and hexane extracts, respectively. According to Köksal et al. [56], the TPC of aqueous and ethanolic extracts of dried thyme (*T. vulgaris*) from Turkey was 256 μg GAE/mg and 158 μg GAE/mg, respectively. Sarfaraz et al. [57] reported TPC values of 35.73 mg GAE/g dw for a methanolic extract obtained of Iranian *T. vulgaris*, which is close to our result for the methanolic extract (31.7 mg GAE/g dw). A study on the antioxidant potential and the bioactive compounds of another thyme species (*T. serpyllum* L.) from the Ladakh plateau, India revealed that the TPC amounted to 86.6 mg GAE/g dw, which was higher than our results for the aqueous, oil, and methanolic thyme extracts but lower than that for the ethanolic one. The authors stated that the antioxidant activity of the aqueous thyme extract showed a value of 48.58 μg/mL determined using the DPPH method [47].

Georgieva et al. [58] investigated five medicinal plants from the western Rhodopes, Bulgaria and determined that the total phenolic content (TPC) of 70% ethanolic extracts of *T. callieri* varied in the range of 20.32 mg GAE/g to 86.19 mg GAE/g for a frozen and a dried herb, respectively. The authors obtained similar results to ours for the TPC of 70% ethanolic extracts of *C. monogyna* fruit, whose values ranged between 13.19 mg GAE/g and 26.88 mg GAE/g for a frozen and a dried herb, respectively. Regarding the antioxidant activity (determined using the DPPH method), the values for *T. callieri* were 121.30 and 218.97 mM TE/g for a frozen and a dried herb and 85.43 and 176.23 mM TE/g for a frozen and a dried *C. monogyna* fruit.

The total phenolic content and antioxidant activity of cotton thistle (*O. acanthium* L.) from Bulgaria were previously examined by Petkova et al. [45]. The authors determined TPC values of 4.06 mg GAE/g dw for the aqueous thistle extract, which is comparable with our result. In contrast, the antioxidant activity of the aqueous thistle extract determined using the DPPH method (22.73 mM TE/g) was higher than our result. Parzhanova et al. [59] investigated the total phenolic content, total flavonoids, and antioxidant potential of cotton thistle (*O. acanthium* L.) from the Rhodope Mountains, Bulgaria. The two ethanolic extracts (50% and 70%) of flowering heads of cotton thistle demonstrated higher TPC (50 mg GAE/g dry extract) in comparison with the aqueous extract. However, the results from the antioxidant activity test demonstrated that the 70% ethanolic extract possessed the highest antiradical value (298.3 mM TE/g) evaluated using the DPPH method, while the aqueous extract exhibited the highest value determined using the FRAP assay. In the same research, the authors reported that the 50% ethanolic extract of flowering heads of cotton thistle originating from the Rhodope Mountains demonstrated the highest TFC (110 mg QE/g dry extract) compared to the aqueous and 70% ethanolic extracts of the same plant.

### 4.3. Flavonoid and Phenolic Acid Content

According to Wang et al. [60], the amount of bioactive compounds such as phenolic acids and flavonoids is affected by genetic variation among species, within the same species, and maturity of plant organs at its harvest. Also, plant phenolic composition may be affected by soil conditions. For example, a higher level of nitrogen and a decrease in soil moisture cause a lower synthesis and, therefore, lower levels of some certain phenolics [61]. On the other hand, light stimulates the synthesis of phenolic compounds such as flavonoids, flavones, and anthocyanins.

Georgieva et al. [58] determined that the total flavonoid content (TFC) of 70% ethanolic extracts of *T. callieri* from the western Rhodopes, Bulgaria varied in the range of 7.47 mg QE/g to 26.43 mg QE/g for a frozen and a dried herb, respectively, while the TFC values of 70% ethanolic extracts of *C. monogyna* fruit from the same geographic location were between 2.27 mg QE/g and 2.89 mg QE/g for a frozen and a dried herb, respectively. In the same research, the authors evaluated the impact of ethanol concentration on the extraction of biologically active compounds and concluded that the highest extraction yield was reached using 70% ethanol (maximum TPC, TFC, and antioxidant activity values using the DPPH and FRAP methods for the frozen and dried herbs, respectively), which is in conformity with our results.

Roby et al. [55] used HPLC to investigate the phenolic content of a methanolic extract of thyme (*T. vulgaris*) from Egypt and identified quinic acid, p-coumaric acid, caffeic acid, rosmaric acid, ferulic acid, carnosic acid, caffeic and ferulic acid derivatives, quercetin-7-o-glucoside, methyl rosmarenate, apigenin, naringnin, and luteolin-7-o-rutinose. The HPLC analysis of Iranian thyme extract performed by Sarfaraz et al. [57] demonstrated the presence of gallic acid (5.5 mg/100 g dw), epicatechin (2.3 mg/100 g dw), caffeic acid (11.8 mg/100 g dw), luteolin-7-o-glucoside (0.8 mg/100 g dw), p-coumaric acid (2.9 mg/100 g dw), ferulic acid (25.6 mg/100 g dw), rosmarinic acid (87.4 mg/100 g dw), salvianolic acid (90 mg/100 g dw), and cinnamic acid (32.3 mg/100 g dw) as the major phenolic acids, as well as apigenin (12.2 mg/100 g dw), naringenin (0.6 mg/100 g dw), and kaempferol (1.1 mg/100 g dw) as the major flavonoid compounds. In comparison, the results for *T. callieri* showed that, in our samples, gallic acid was not detected, while caffeic acid, rosmarinic acid, apigenin, and kaempferol were found in higher amounts. According to Asl et al. [62], this difference in the concentration of the phenolic components in plants is the result of some environmental factors. Wang et al. [63] reported that the higher flavonoid content and concentrations of phenolic compounds result from growing temperatures and levels of CO_2_.

Yanar et al. [51] analysed wild hawthorn fruit (*C. monogyna*) belonging to three different genotypes and found the following phenolic compounds varying in different amounts: gallic acid (0.32–1.61 mg/100 g fw), catechin (15.1–143.82 mg/100 g fw), epicatechin (1.32–35.09 mg/100 g fw), rutin (6.62–21.82 mg/100 g fw), epigallocatechin gallate (0.75–79.5 mg/100 g fw), and caffeic acid (0.01–1.03 mg/100 g fw). Our results on hawthorn fruit showed that gallic acid was not detected, while the caffeic acid amount was higher (9.4 mg/100 g dw). Other authors have also detected chlorogenic acid in hawthorn fruit [18,64], as well as caffeic acid and ellagic acid [65]. The antioxidant activity of hawthorn fruit determined using two methods showed values of 280.7 μmol TE/g (by ORAC) and 107.5 μmol GAE/g (by HORAC) [65]. Khokhlova et al. [66] identified caffeic acid, chlorogenic acid, oleanolic acid, ursolic acid, hyperoside, vitexin, vitexin 2-rhamnoside, vitexin 2-O-rhamnoside, rutin, and naringenin in hawthorn fruit. In contrast to our results, which showed the presence of quercetin-3-β-glucoside and myricetin only, Keser et al. [67] identified the flavonoids rutin, apigenin, myricetin, quercetin, naringenin, and kaempferol.

### 4.4. Antimicrobial Activity

The medicinal plants are rich sources of biologically active compounds that determine their pharmacological and beneficial health effects such as antimicrobial activity. The development of new drugs based on plants possessing antimicrobial potential could be a promising approach for solving the problem of antibiotic resistance. In this regard, the extracts of the three investigated plants could be successfully used as antimicrobial agents or ingredients in medical formulations.

Some authors such as Al-Juraifani [68] evaluated plants from Saudi Arabia and found that an ethanolic–water extract of thyme (*T. vulgaris*) exhibited the highest antimicrobial activity against *Streptococcus* sp., *S. aureus*, *Vibrio tubiashii*, *M. luteus* ATCC 9341, *Cellulosimicrobium cellulans*, *B. cereus*, *Legionella pneumophila,* and two fungal species, *A. flavus* and *Fusarium oxysporum*, and that the inhibitory activity increased with heightened concentrations of the plant extracts. Mokhtari et al. [69] observed high antimicrobial activity of a methanolic extract of thyme (*T. vulgaris*) against *B. cereus* with an inhibition zone (IZ) 24.87 mm in diameter, *S. aureus* (IZ = 23.56 mm), *E. coli* (IZ = 18.43 mm), and *S. typhimurium* (IZ = 17.11 mm) and concluded that the investigated extract could be successfully applied as an antibacterial agent. According to the same authors, the antibacterial activity of thyme was due to the presence of the biologically active compounds thymol, cinnamic acid, and carvacrol, which increased the permeability of the cell membrane, inhibited the bacterial enzymes, and disrupted the synthesis of the cell structural components or the genetic substance, especially in the gram-positive bacteria.

The fruits and leaves of *C. monogyna* are characterised by high antimicrobial activity, mainly displayed against gram-positive bacteria [70]. A study performed by Tadić et al. [22] on the antimicrobial activity of an ethanolic extract of hawthorn fruit at a concentration of 10 mg/mL showed that the extract had a strong inhibitory effect on *Micrococcus flavus* and *B. subtilis*, a moderate inhibitory effect on *L. monocytogenes*, *Streptococcus epidermidis*, *M. luteus*, and *P. aeruginosa* and a weak effect on *E. coli*, *S. aureus,* and *C. albicans* yeast. Yiğit et al. [71] examined aqueous and methanolic extracts of hawthorn fruit and leaves against clinical isolates of human pathogenic strains (*Enterobacter aerogenes*, *S. aureus, E. coli*, and *P. aeruginosa* and yeasts *C. albicans*, *Candida glabrata,* and *Candida parapisilosis*) and stated that both extracts possessed limited antimicrobial activity. The methanolic hawthorn fruit extract exhibited low inhibitory activity against *S. aureus*, whereas the aqueous hawthorn fruit extract had no inhibitory activity against the microorganisms tested. Pugna et al. [72] found that the inner layers of hawthorn fruit possessed high inhibitory activity against *E. coli* ATCC 25922 (IZ = 40 mm) and *P. aeruginosa* ATCC 27852 (IZ = 30 mm) but did not inhibit the pathogen *S. aureus* ATCC 25923.

Few studies about the antimicrobial activity of *O. acanthium* have been reported previously. Zare et al. [73] found that a methanolic extract of cotton thistle seeds displayed significant antibacterial activity against *Staphylococcus epidermidis* (IZ = 18.66 mm) and *M. luteus* (IZ = 21 mm) but had no inhibitory effect on *E. coli*, *S. aureus,* and *K. pneumoniae*. Móricz et al. [74] studied the antimicrobial effect of *O. acanthium* L. leaf extract and, using HPLC analysis, identified the three compounds responsible for its antibacterial activity: linoleic, linolenic acid, and germacranolide sesquiterpene lactone (onopordopicrin).

## 5. Conclusions

Based on the estimations of the present research, we can conclude that the three herbs, thyme, hawthorn fruit, and cotton thistle, from the western Rhodope Mountains, Bulgaria are an excellent source of nutrients and compounds with promising antioxidant activity (biological potential factors), which might be used in the food industry as food ingredients and food enhancers or in the pharmaceutical industry as active components of different medical formulations. In view of the antimicrobial potential of the investigated herb extracts, they can find successful practical applications in biopreservation and the extension of the shelf life of various food products.

## Figures and Tables

**Table 1:** Origin of the three Bulgarian medicinal plants.

Plant	Region	District	GPS Coordinates	Altitude, m
Thyme (*Thymus callieri* Borbás ex Velen.)	Near Dospat	Smolyan	41°66′ N 24°16′ E	1214
Cotton thistle (*Onopordum acanthium* L.)	Dospat, Chillii locality	Smolyan	41°66′ N 24°16′ E	1207
Hawthorn (*Crataegus monogyna* Jacq.)	Satovcha, Aspen locality	Blagoevgrad	41°63′N 24°51′ E	1134

**Table 2:** Standards for determination of the physicochemical characteristics.

Parameter	Standard
Moisture	BSS ISO 939:2021 [35]
Ash	BSS ISO 928:2004 [36]
Carbohydrates	BSS 7169:1989 [37]
Proteins	BSS 15438:1982 [38]
Vitamin C	BSS 11812:1991 [39]

**Table 3:** Physicochemical characteristics of thyme, cotton thistle, and hawthorn fruit.

Parameter	Thyme	Cotton Thistle	Hawthorn Fruit
Moisture, %	8.15 ± 0.25 ^ab^	7.53 ± 0.24 ^b^	8.50 ± 0.25 ^a^
Ash, %	6.62 ± 0.01 ^a^	4.91 ± 0.01 ^b^	2.62 ± 0.00 ^c^
Carbohydrates, %	3.20 ± 0.22 ^b^	0.60 ± 0.06 ^c^	4.20 ± 0.22 ^a^
Proteins, %	11.30 ± 0.73 ^a^	8.33 ± 0.55 ^b^	2.95 ± 0.26 ^c^
Vitamin C, mg/100 g	571.00 ± 2.02 ^a^	407.00 ± 1.35 ^c^	430.00 ± 1.57 ^b^

^a–c^: means in a row without a common letter differ significantly (*p* ≤ 0.05).

**Table 4:** Total phenolic content and antioxidant activity of the thyme, cotton thistle, and hawthorn fruit extracts determined using the DPPH method.

Plant Extract	Total Polyphenols, mg GAE/g				Antioxidant Activity			
					DPPH, μmol TE/g			
	Aqueous	Oil	MeOH	EtOH	Aqueous	Oil	MeOH	EtOH
Thyme	27.25 ± 0.61 ^b,A^	8.20 ± 0.13 ^c,A^	31.70 ± 0.75 ^b,A^	310.00 ± 4.25 ^a,A^	35.93 ± 0.45 ^c,A^	15.66 ± 0.21 ^d,C^	320.75 ± 3.39 ^b,A^	397.48 ± 2.98 ^a,A^
Cotton thistle	6.20 ± 0.05 ^c,C^	5.30 ± 0.02 ^c,B^	14.90 ± 0.31 ^b,B^	168.00 ± 2.05 ^a,B^	5.26 ± 0.03 ^c,C^	19.93 ± 0.18 ^b,B^	4.67 ± 0.04 ^c,B^	45.00 ± 1.12 ^a,B^
Hawthorn fruit	12.80 ± 0.14 ^b,B^	3.70 ± 0.00 ^d,C^	9.20 ± 0.08 ^c,C^	13.60 ± 0.11 ^a,C^	11.28 ± 0.15 ^c,B^	21.80 ± 0.23 ^b,A^	2.09 ± 0.01 ^d,C^	38.99 ± 0.89 ^a,C^

^a–d^: means in a row (for a method) without a common letter differ significantly (*p* ≤ 0.05). ^A–C^: means in a column (for an extraction method) without a common letter differ significantly (*p* ≤ 0.05).

**Table 5:** Antioxidant activity of the thyme, cotton thistle, and hawthorn fruit extracts determined using the ORAC and HORAC methods.

Plant Extract	Antioxidant Activity							
	ORAC, µmol TE/g				HORAC, µmol GAE/g			
	Aqueous	Oil	MeOH	EtOH	Aqueous	Oil	MeOH	EtOH
Thyme	1483.08 ± 10.3 ^b,A^	29.63 ± 1.11 ^d,A^	458.98 ± 3.14 ^c,A^	3221.95 ± 41.92 ^a,A^	426.72 ± 6.91 ^b,A^	n.d.	170.19 ± 6.09 ^c,A^	961.21 ± 2.36 ^a,A^
Cotton thistle	241.05 ± 0.43 ^b,C^	11.41 ± 0.56 ^d,B^	117.49 ± 1.33 ^c,C^	1742.49 ± 33.80 ^a,C^	48.93 ± 2.74 ^b,C^	n.d.	42.13 ± 0.82 ^b,C^	716.37 ± 6.75 ^a,B^
Hawthorn fruit	469.51 ± 0.81 ^b,B^	10.30 ± 0.52 ^d,B^	177.13 ± 5.99 ^c,B^	1891.73 ± 12.67 ^a,B^	173.05 ± 6.22 ^b,B^	n.d.	65.24 ± 7.06 ^c,B^	669.83 ± 1.39 ^a,C^

^a–d^: means in a row (for a method) without a common letter differ significantly (*p* ≤ 0.05). ^A–C^: means in a column (for an extraction method) without a common letter differ significantly (*p* ≤ 0.05).

**Table 6:** Flavonoid content of thyme, cotton thistle, and hawthorn fruit determined using HPLC.

70% EtOH Extracts	Quercetin-3-β- Glucoside, mg/100 g	Myricetin, mg/100 g	Kaempferol, mg/100 g	Apigenin, mg/100 g	Luteolin, mg/100 g
Thyme	374.5 ± 4.0	-	16.1 ± 0.1	16.4 ± 0.4	73.8 ± 0.2
Cotton thistle	-	152.3 ± 0.3	42.2 ± 0.1	85.1 ± 0.2	-
Hawthorn fruit	48.5 ± 0.1	10.9 ± 0.2	-	-	-

**Table 7:** Phenolic acid content of thyme, cotton thistle, and hawthorn fruit determined using HPLC analysis.

70% EtOH Extracts	Neochlorogenic Acid, mg/100 g	Chlorogenic Acid, mg/100 g	Gallic Acid, mg/100 g	Rosmaric Acid, mg/100 g	Caffeic Acid, mg/100 g
Thyme	-	-	-	995.0 ± 0.6	26.0 ± 1.6
Cotton thistle	19.2 ± 0.1	324.0 ± 4.4	5.8 ± 0.1	-	-
Hawthorn fruit	12.3 ± 0.5	27.7 ± 0.1	-	-	9.4 ± 0.1

**Table 8:** Antibacterial activity of thyme, cotton thistle, and hawthorn fruit extracts.

Test Microorganism	Inhibition Zones (IZ), mm											
	Aqueous			MeOH			70% EtOH			Controls (10 mg/mL)		
	T *	HF **	CT ***	T *	HF **	CT ***	T *	HF **	CT ***	Amp.	Pen.	Cypr.
*B. subtilis* ATCC 6633	-	10	-	12	10	11	14	14	20	19	18	32
*B. amyloliquefaciens* 4BCL-YT	-	-	-	10	8	10	13	9	-	30	32	30
*S. aureus* ATCC 25923	10	-	-	9	-	-	18	-	17	34	36	35
*L. monocytogenes* NBIMCC 8632	13	13	13	9	-	-	14	12	13	30	28	21
*E. faecalis* ATCC 29212	-	13	13	8	-	-	12	10	13	30	30	30
*M. luteus* 2YC-YT	20	15	22	10	9	9	17	16	16	33	33	33
*S. enteritidis* ATCC 13076	12	-	12	12	-	-	13	10	13	35	30	36
*S. typhimurium* NBIMCC1672	-	-	-	12	10	11	11	11	11	14	19	33
*K. pneumoniae* ATCC 13883	-	-	-	14	14	10	14	11	10	19	21	29
*E. coli* ATCC 25922	-	8	-	12	10	10	18	15	18	18	18	30
*P. vulgaris* ATCC 6380	-	-	-	13	12	12	12	11	8	25	19	28
*lP. aeruginosa* ATCC 9027	-	8	-	13	10	12	17	15	16	23	20	33

* T—Thyme extract; ** HF—Hawthorn fruit extract; *** CT—Cotton thistle extract; d_well_ = 6 mm. Controls: Amp.—Ampicillin; Pen.—Penicillin; and Cypr.—Cyprofloxacin.

**Table 9:** Antifungal activity of thyme, cotton thistle, and hawthorn fruit extracts.

Test Microorganism	Inhibition Zones (IZ), mm											
	Aqueous			MeOH			70% EtOH			Controls (10 mg/mL)		
	T *	HF **	CT ***	T *	HF **	CT ***	T *	HF **	CT ***	Fluc.	N. (M)	N. (W)
*C. albicans* NBIMCC 74	-	-	-	8	8	8	-	-	8	-	35	20
*S. cerevisiae* ATCC 9763	-	-	-	8	-	-	8	-	8	-	30	20
*A. niger* ATCC 1015	-	-	-	8	-	-	-	-	8	15	35	23
*A. flavus*	-	-	-	-	-	-	-	-	-	-	30	16
*P. chrysogenum*	10	11	15	10	10	8	8	-	8	-	35	13
*Rhizopus* sp.	11	12	10	8	-	-	8	-	8	-	32	16
*F. moniliforme* ATCC 38932	-	-	-	8	-	-	8	-	8	-	29	15
*Mucor* sp.	-	-	-	-	-	-	-	-	-	-	35	18

* T—Thyme extract; ** HF—Hawthorn fruit extract; *** CT—Cotton thistle extract; d_well_ = 6 mm. Controls: Fluc.—Fluconazole; N. (M)—Nystatin diluted in methanol; and N. (W)—Nystatin diluted in distilled water.

## Data Availability

Data is contained within the article.
